# American Medical Association

**Published:** 1871-06

**Authors:** 


### AMERICAN MEDICAL ASSOCIATION.

FIRST DAY.

The Twenty-Second Annual Meeting of the American Medical Association, organized in Philadelphia in 1847, was commenced at Pacific Hall, on May 2, '71. About 200 medical gentlemen were present. The present officers are:—President, Dr. Alfred Stillé, of Pennsylvania; Vice-Presidents, Dr. J. S. Wetherly, of Alabama; Dr. Henry Gibbons, of California; Dr. T. J. Heard, of Texas; Dr. Samuel Willey, of Minnesota; Permanent Secretary, W. B. Atkinson, M.D., Philadelphia; Assistant-Secretary, Dr. Joseph Tucker, of California; Treasurer, Dr. Caspar Wistar, of Pennsylvania; Librarian, Dr. F. A. Ashford, of District of Columbia.

About half-past eleven o'clock, Dr. Arthur B. Stout called the meeting to order, and introduced President Dr. A. Stillé, who was greeted with applause.

The Right Rev. Bishop Kip was next introduced, and offered a prayer to the Throne of Grace.

The Committee of Arrangements were called on for a report of the credentials of members.

Dr. Stout came forward, and in some appropriate remarks greeted the members of the Association from the East. He spoke of the scientific interests and the progress they had made in the last quarter of a century. He next referred to the improvement and rapid strides made in California in all departments of learning, science, and art, and then adverted to the many obstacles thrown in the way of the medical profession, and the attacks made on it by the bar, ministry, poets, and authors. In conclusion, he said it was proposed to give the members an opportunity to see some of the sights of this city and vicinity.

Dr. Stout reported that the registration had not yet been completed, 200 members having thus far been registered.

On motion, the Committee were given until the following day to present their report.

A letter was read from Prof. S. D. Gross, of Philadelphia, ex-President of the Association, regretting his inability to attend the sessions of the Association. It was ordered spread on the minutes.

The Secretary read invitations from the California Pioneers, tendering the freedom of their hall to the members of the Association, and from the Board of Health of the city, inviting the

Association to visit the City and County Hospital, Alms House, Industrial School, City Prison, County Jail, and Health Office. The invitations were received and filed.

On motion of Dr. Stout, all members of the California State Medical Society not delegates were invited to sit as members by invitation.

